# Editorial Note: A study of systemic risk spillovers in Asian emerging markets and Chinese stock market

**DOI:** 10.1371/journal.pone.0349182

**Published:** 2026-05-12

**Authors:** The PLOS One Editors

The *PLOS One* Editors issue this Editorial Note to inform readers that the work cited in this article [1] as Reference 49 was retracted after the article’s publication date. PLOS considers that this reference is not crucial in supporting the research or conclusions reported in [1], but the following sentence in the Empirical results section is no longer supported: In contrast, later in the pandemic, risk spillovers from China’s SSE intensified toward export-oriented economies such as South Korea (KOSPI) and Taiwan (TWII) [49].
